# The roles of inactivated vaccines in older patients with infection of Delta variant in Nanjing, China

**DOI:** 10.18632/aging.204085

**Published:** 2022-05-18

**Authors:** Xiao-Chun Song, Xue-Hui Zhou, Jing-Hui Cheng, Wen-Hao Zhang, Xiao Shen, Huan Xu, Shuai Nie, Ji-Lai Xiao, Fang Sun, Chang Shu, Jiu-Dong Chen, Yan Tang, Xiang Wang, Xin-Pei Sun, Jia-Kui Sun, Ping Feng, Qian-Kun Shi

**Affiliations:** 1 Department of Critical Care Medicine, Nanjing First Hospital, Nanjing Medical University, Nanjing 210006, Jiangsu Province, China; 2 Department of General Office, Productivity Center of Jiangsu Province, Nanjing 210042, Jiangsu Province, China

**Keywords:** inactivated vaccines, organ dysfunction, older patients, Delta variant, COVID-19

## Abstract

Background: The coronavirus disease 2019 (COVID-19) is spreading around the world. The COVID-19 vaccines may improve concerns about the pandemic. However, the roles of inactivated vaccines in older patients (aged ≥60 years) with infection of Delta variant were less studied.

Methods: We classified the older patients with infection of Delta variant into three groups based on the vaccination status: no vaccination (group A, *n* = 113), one dose of vaccination (group B, *n* = 46), and two doses of vaccination (group C, *n* = 22). Two inactivated COVID-19 vaccines (BBIBP-CorV or CoronaVac) were evaluated in this study. The demographic data, laboratory parameters, and clinical severity were recorded.

Results: A total of 181 older patients with infection of Delta variant were enrolled. 111 (61.3%) patients had one or more co-morbidities. The days of "turn negative" and hospital stay in Group C were lower than those in the other groups (*P* < 0.05). The incidences of multiple organ dysfunction syndrome (MODS), septic shock, acute respiratory distress syndrome (ARDS), acute kidney injury, and cardiac injury in Group A were higher than those in the other groups (*P* < 0.05). The MV-free days and ICU-free days during 28 days in Group A were also lower than those in the other groups (*P* < 0.05). In patients with co-morbidities, vaccinated cases had lower incidences of MODS (*P* = 0.015), septic shock (*P* = 0.015), and ARDS (*P* = 0.008).

Conclusions: The inactivated COVID-19 vaccines were effective in improving the clinical severity of older patients with infection of Delta variant.

## INTRODUCTION

The coronavirus disease 2019 (COVID-19) continues to spread throughout all parts of the world [1–3], and more and more mutant variants have exacerbated the global pandemic [2, 4], which may also facilitate escape from vaccine protection and current therapies in unexpected ways [5, 6].

In addition to the nonpharmaceutical interventions and symptomatic treatments, new SARS-CoV-2 vaccines may improve concerns about the global pandemic [7]. Several inactivated vaccines against SARS-CoV-2 (e.g., ZF2001, CoronaVac, BBIBP-CorV) have been demonstrated to be generally effective and safe in large sample clinical studies [7–9]. Besides that, these vaccines are also well-tolerated in 60 years and older adults and could reduce the severity of COVID-19 [7, 10]. However, elderly people with co-morbidities or frailty were usually not included in the previous phase 1 to 3 trials [11]. In both CD8 and CD4 cells, aging has been shown to result in a reduction of T cell receptor diversity, which may lead to reduced T cell survival [11]. Aging could also decrease the production of functional antibodies because of reduced expression of select proteins [11]. Hence, the current vaccines may be theoretically ineffective in older people.

An imported COVID-19 infection related to the Delta strain (the B.1.617.2 variant) erupted in the Chinese city of Nanjing on July 21, 2021 [3, 12]. Considering the mutating variants, the effectiveness of various types of vaccines should also be confirmed by more studies. While conducting our clinical effort to combat the COVID-19 epidemic in Nanjing, we discovered that there were a number of older and vaccinated patients among confirmed cases. Therefore, we aimed to investigate the roles of inactivated SARS-CoV-2 vaccines in older patients with infection of Delta variant, especially in those with co-morbidities.

## METHODS

### Patients

From July 21 to September 13, 2021, older patients (age ≥60 years) with confirmed infection of Delta variant admitted to specialized isolation units, Nanjing Public Health Center (Nanjing Second Hospital), were recruited to participate in this clinical retrospective study. The only hospital in Nanjing that treated COVID-19 patients was the Nanjing Public Health Center. All the older patients were classified as high-risk groups for severe or critical [12]. Therefore, these patients received grade one (in the ward) or special (in ICU) nursing care in our specialized isolation units. Our institutional review board waived written informed consent since this was retrospective research that gathered de-identified data with no possible danger to the patients. The COVID-19 (Delta variant) was diagnosed in accordance with the guidelines of the National Health Commission (NHC) of China and WHO [3, 12], and verified via RNA test of SARS-CoV-2 in the specialized lab for clinical research in Nanjing Second Hospital. The vaccination recommendations followed the COVID-19 vaccination technical guidelines of the NHC of China [12]. Two inactivated SARS-CoV-2 vaccines (BBIBP-CorV or CoronaVac) were available in Nanjing city before and during the study period. Two doses of the inactivated vaccines were recommended, with an interval of 3 to 8 weeks [12].

### Definitions

The clinical classification of COVID-19 was recommended by the NHC of China [12, 13]: Mild, with minor clinical signs (such as fever and cough) and no imaging manifestations. Moderate, with indications of respiratory tract infections and pneumonia-like imaging characteristics. Severe, having satisfied one or more of the conditions below: (1) respiratory discomfort and a breathing rate of more than 30 breaths per minute; (2) At rest, the pulse oxygen saturation (SpO2) is less than or equal to 93 percent; (3) arterial partial pressure of oxygen (PaO2)/ fraction of inspired oxygen (FiO2) ≤300 mmHg (1 mmHg = 0.133 kPa). Critical, having satisfied one of the criteria below: (1) respiratory failure accompanied by mechanical ventilation (MV); (2) shock; (3) admission into the ICU as a result of multiple organ dysfunction. Sepsis was described as fatal organ failure produced by a dysfunctional host defense against pathogens, whereas septic shock was described as a subtype of sepsis characterized by metabolic/cellular and circulatory impairment that is linked to a greater risk of death [14, 15].

The Berlin standards for acute respiratory distress syndrome (ARDS) were used in making the diagnosis [16]. The presence of liver damage was determined when the serum concentrations of hepatic biological markers (e.g., alanine aminotransferase) exceeded twice the reference upper limit, or when there was an abnormally elevated level of aspartate aminotransferase (AST) and alanine aminotransferase (ALT) when especially in comparison with alkaline phosphatase levels [17]. It was determined that the patient had acute kidney injury (AKI) in accordance with the 2012 Kidney Disease: Improving Global Outcomes (KDIGO) guidelines [18]. An increase in blood levels of biological markers (e.g., troponin I), exceeding twice the reference upper limit, or the discovery of new aberrations in echocardiography and electrocardiography, was considered evidence of cardiac damage [17]. During the 365-day period before admission to the hospital, comorbidity was considered as present when there was at least 1 specific procedure or 2 specific outpatient procedures, or a prescription for a medicine that characterized the comorbid condition in the 365-day period. Multiple organ dysfunction syndromes (MODS) are recognized as the simultaneous malfunctions of two or more organs that have been identified in an individual.

### Data collection

The baseline clinical features, which included body mass index (BMI), age, and sex, days from occurrence to hospitalization, days from vaccination to admission, days from onset to SARS-CoV-2 testing negative (days of “turn negative”), early signs and symptoms, clinical classifications, and co-morbidities, were obtained. All of the information was derived from electronic medical records, which had to be manually retrieved and the information of each patient was also checked by another investigator. The serum levels of lymphocyte count, white blood cell (WBC) count, ALT, C-reactive protein (CRP), creatinine, D-dimer, brain natriuretic peptide (BNP), procalcitonin (PCT), troponin I (TNI), and interleukin-6 (IL-6) were obtained upon admission. The serum levels of percentages of CD4 and CD8 lymphocytes, virus immunoglobulin (Ig) M and IgG antibody, and the cycle threshold (CT) of RT-PCR assays of admission were also acquired. The professional clinical laboratory of Nanjing Second Hospital was responsible for detecting all of the hematological parameters.

The number of patients with septic shock, cardiac injury, AKI, MODS, liver damage, and ARDS, and patients requiring high-flow nasal cannula (HFNC) or noninvasive ventilation (NIV), continuous renal replacement therapy (CRRT), MV, or extracorporeal membrane oxygenation (ECMO) were collected. The thromboembolic events (e.g., cerebral infarction, cerebral infarction, venous thromboembolism) were also counted. The length of NIV or HFNC, length of hospital stay (LOS), MV-free days, and ICU-free days within the initial 28 days and the 28-day mortality were also collected.

### Statistical analysis

The Kolmogorov-Smirnov test was the first to be performed to evaluate the normal distribution of data. Data with normal distributions were presented as the means ± standard deviation with comparisons made using *t*-tests. Data with abnormal distributions were presented as the medians (interquartile ranges, IQR) with comparisons made with the help of the Kruskal-Wallis test and Mann-Whitney *U* test. In this study, categorical data were reported as percentages or absolute numbers, and they were evaluated utilizing the Fisher’s exact or χ^2^ test. Additionally, we performed an analysis of variance (ANOVA) for multiple testing of the general linear model in order to take into consideration the repetitiveness of the variables. The analysis of statistical data was carried out utilizing IBM Statistical Package for the Social Sciences (SPSS, version 22.0, New York, USA) program, and *P* < 0.05 was established as the criterion of statistical significance. Qiao Liu, a biostatistician from the Jiangsu Provincial Center for Disease Control and Prevention in China, examined the statistical techniques used in this research.

## RESULTS

During the course of this clinical retrospective analysis, 181 older individuals with verified COVID-19 (Delta variant) infection were included. The median age was 69 (interquartile range, 65.5–74) years, with 107 (59.1 percent) of the participants being female. Among these patients, 113 (62.4%) were not vaccinated, 46 (25.4%) received one dose of vaccine, and only 22 (12.2%) received two doses of vaccine. One hundred and forty-five (80.1%) patients were categorized as moderate, 21 (11.6%) patients were categorized as severe, and 15 (8.3%) patients were categorized as critical. One hundred and eleven (61.3%) patients had one or more co-morbidities. MODS occurred in 14 patients (7.7% of the total), while septic shock occurred in 12 individuals (6.7% of the total). Two (1.1%) critically ill patients died within 28 days of admission. Table 1 contained the comprehensive clinical information of the patients.

**Table 1:** Demographic data and clinical parameters (*n* = 181).

**Variables**	**Values**
Age (years)	69 (65.5–74)
Sex (Male: Female)	74:107
BMI (kg/m^2^)	23.7 (22.2–26.6)
Days from onset to admission	3 (2–5)
Days from vaccination to admission	14 (8–24)
Days of “turn negative”	23 (18–27)
Initial symptoms or signs (*n*, %)	
Fever	60 (33.1%)
Cough	47 (26.0%)
Fatigue	19 (10.5%)
Pharyngalgia	10 (5.5%)
Headache or dizziness	8 (4.4%)
Stuffy nose	6 (3.3%)
Chest tightness or pain	6 (3.3%)
Anorexia	5 (2.8%)
Diarrhea	5 (2.8%)
Nausea or vomiting	4 (2.2%)
Myalgia	3 (1.7%)
Other	8 (4.4%)
Classifications (*n*, %)	
Mild	0 (0%)
Moderate	145 (80.1%)
Severe	21 (11.6%)
Critical	15 (8.3%)
Co-morbidities (repeated)	
Hypertension	82 (45.3%)
Diabetes mellitus	28 (15.5%)
Chronic respiratory diseases	15 (8.3%)
Coronary heart disease	14 (7.7%)
Cerebral infarction	8 (4.4%)
Chronic liver or kidney disease	3 (1.7%)
Other	5 (2.8%)
Blood parameters	
CRP (mg/L)	11.6 (3.4–30.6)
WBC (10^9^/L)	4.7 (3.8–6.2)
Lymphocyte (10^9^/L)	1.0 (0.8–1.4)
ALT (U/L)	20.5 (15.1–32.1)
Creatinine (umol/L)	64.3 (55.2–78.9)
TNI (pg/mL)	5.6 (1.6–12.4)
D-dimer (mg/L)	0.5 (0.3–0.7)
BNP (pg/mL)	24 (12.2–56.2)
PCT (ng/mL)	0.1 (0.0–0.1)
IL-6 (pg/mL)	24.2 (12.1–37.9)
CD4 T cells percentage (%)	39.0 (33.0–44.5)
CD8 T cells percentage (%)	21 (17–25)
IgM antibody (S/CO)	0.1 (0–0.5)
IgG antibody (S/CO)	0.2 (0.1–1.3)
PCR cycle threshold (CT values)	
ORF1ab gene	23 (20–26)
N gene	20 (17–24)
Organs injury (*n*, %)	
ARDS	15 (8.3%)
Liver injury	11 (6.1%)
AKI	11 (6.1%)
Cardiac injury	12 (6.7%)
MODS	14 (7.7%)
Thrombo-embolic events (*n*, %)	0 (0%)
Septic shock (*n*, %)	12 (6.7%)
Need for NIV/HFNC (*n*, %)	33 (18.2%)
Need for MV (*n*, %)	15 (8.3%)
Need for CRRT/ECMO (*n*, %)	6 (3.3%)
NIV/HFNC days	1.2 ± 3.0
MV-free days	26.4 ± 5.6
ICU-free days	25.4 ± 6.8
Hospital stay (days)	26 (21–30)
Death (*n*, %)	2 (1.1%)

Abbreviations: BMI: body mass index; CRP: C-reactive protein; WBC: white blood cells; ALT: alanine aminotransferase; TNI: troponin I; BNP: brain natriuretic peptide; PCT: procalcitonin; IL-6: interleukin-6; IgM: immunoglobulin M; IgG: immunoglobulin G; PCR: polymerase chain reaction; ARDS: acute respiratory distress syndrome; AKI: acute kidney injury; MODS: multiple organ dysfunction syndrome; NIV: noninvasive ventilation; HFNC: high-flow nasal cannula; MV: mechanical ventilation; CRRT: continuous renal replacement therapy; ECMO: extracorporeal membrane oxygenation; ICU: intensive care unit.

We classified the patients into three groups on the basis of their vaccination status: no vaccination (group A, *n* = 113), one dose of vaccination (group B, *n* = 46), and two doses of vaccination (group C, *n* = 22). As described in Table 2, the days from vaccination to admission in Group B were considerably lower as opposed to those in Group C (*P* = 0.035). The days of "turn negative" and hospital stay in Group C were substantially reduced as opposed to those in Group A or Group B (*P* < 0.05). The serum levels of TNI, BNP, and PCT in Group C were remarkably reduced as opposed to those in Group A or Group B (*P* < 0.05). The serum TNI and BNP levels in Group B were also reduced in contrast with those in Group A (*P* < 0.05). The levels of virus IgM and IgG antibodies in Group C were considerably elevated as opposed to those in Group A or Group B (*P* < 0.05). The levels of virus IgM and IgG antibodies in Group B were also substantially elevated in contrast with those in Group A (*P* < 0.05).

**Table 2:** The clinical parameters and severity variables.

	**Group A (*n* = 113)**	**Group B (*n* = 46)**	**Group C (*n* = 22)**	***P* value**
Days from onset to admission	3.0 (2.0–5.0)	4.0 (2.0–5.3)	3.5 (1.8–6.0)	0.701
Days from vaccination to admission	/	12.5 (8.0–20.0)	30.0 (8.0–44.5)	0.035
Days of “turn negative”	23.0 (18.0–27.0)	22.5 (18.0–26.0)	17.0 (13.8–23.3)	0.026
CRP (mg/L)	10.4 (3.4–28.1)	21.1 (4.0–33.9)	6.5 (2.1–26.9)	0.237
WBC (10^9^/L)	4.5 (3.5–6.0)	5.0 (4.3–6.2)	5.0 (4.1–6.2)	0.156
Lymphocyte (10^9^/L)	1.0 (0.8–1.4)	1.0 (0.7–1.5)	1.1 (0.9–1.5)	0.775
ALT (U/L)	21.2 (15.6–31.1)	19.0 (14.2–32.3)	20.3 (15.1–32.9)	0.638
Creatinine (umol/L)	61.8 (55.3–79.1)	71.8 (55.0–81.7)	60.4 (54.1–73.4)	0.312
TNI (pg/mL)	6.1 (2.2–18.9)	4.1 (1.0–8.3)	3.8 (1.8–9.6)	0.041
D-dimer (mg/L)	0.5 (0.4–0.8)	0.5 (0.3–0.7)	0.5 (0.3–0.7)	0.507
BNP (pg/mL)	29.2 (12.6–65.3)	20.5 (12.0–44.8)	17.4 (10.0–34.6)	0.039
PCT (ng/mL)	0.06 (0.04–0.1)	0.06 (0.04–0.1)	0.03 (0.02–0.06)	0.043
IL-6 (pg/mL)	25.7 (12.9–39.7)	21.9 (12.6–33.4)	16.3 (8.1–32.7)	0.088
CD4 percentage (%)	38.0 (32.0–43.0)	41.0 (33.8–46.0)	42.0 (37.8–46.3)	0.109
CD8 percentage (%)	21.0 (18.0–25.0)	20.0 (16.8–25.3)	22.0 (16.8–25.5)	0.674
IgM (S/CO)	0.06 (0.03–0.3)	0.2 (0.1–0.8)	0.6 (0.2–2.3)	<0.001
IgG (S/CO)	0.1 (0.06–0.3)	0.3 (0.1–12.5)	10.9 (3.4–104.8)	<0.001
CT values of ORF1ab gene	22.0 (19.0–26.0)	24.0 (20.8–26.0)	22.0 (18.8–25.3)	0.400
CT values of N gene	20.0 (17.0–24.0)	20.0 (17.0–24.0)	21.0 (16.8–24.0)	0.921

Abbreviations: Group A: No vaccination; Group B: One dose of vaccination; Group C: Two doses of vaccination; CRP: C-reactive protein; WBC: white blood cells; ALT: alanine aminotransferase; TNI: troponin I; BNP: brain natriuretic peptide; PCT: procalcitonin; IL-6: interleukin-6; IgM: immunoglobulin M; IgG: immunoglobulin G; CT: cycle threshold.

Table 3 highlighted the differences in clinical severity and outcome characteristics across the 3 groups. The incidences of MODS, septic shock, ARDS, AKI, cardiac injury, and other complications in Group A were remarkably elevated as opposed to the ones in Group B or Group C (*P* < 0.05). The proportions of patients receiving HFNC/NIV or MV in Group A were also considerably increased compared to those in Group B or Group C (*P* < 0.05). However, no differences in the abovementioned parameters were discovered between Group B and Group C (*P* > 0.05). The ICU-free and MV-free days within the initial 28 days in Group A were dramatically reduced in contrast with those in Group B or Group C (*P* < 0.05). No difference was identified in the 28-day mortality among the three groups (*P* = 0.315). The above findings illustrated that patients with two doses of vaccination may have a lower incidence of organ injury and less requirement for supportive treatments in ICU.

**Table 3:** Clinical variables of severity and outcomes.

	**Group A (*n* = 113)**	**Group B (*n* = 46)**	**Group C (*n* = 22)**	***P* value**
MODS (*n*, %)	14 (12.4%)	0 (0%)	0 (0%)	0.006
Septic shock (*n*, %)	12 (10.6%)	0 (0%)	0 (0%)	0.011
ARDS (*n*, %)	15 (13.3%)	0 (0%)	0 (0%)	0.004
Liver injury (*n*, %)	8 (7.1%)	2 (4.3%)	1 (4.5%)	0.516
AKI (*n*, %)	10 (8.8%)	1 (2.2%)	0 (0%)	0.048
Cardiac injury (*n*, %)	11 (9.7%)	1 (2.2%)	0 (0%)	0.035
Other complications (*n*, %)	15 (13.3%)	0 (0%)	0 (0%)	0.004
Need for NIV/HFNC (*n*, %)	26 (23.0%)	6 (13.0%)	1 (4.5%)	0.022
Need for MV (*n*, %)	15 (13.3%)	0 (0%)	0 (0%)	0.004
Need for CRRT/ ECMO (*n*, %)	6 (5.3%)	0 (0%)	0 (0%)	0.079
NIV/HFNC days	1.4 ± 3.0	1.2 ± 3.5	0.3 ± 1.5	0.096
MV-free days	25.5 ± 6.9	28 ± 0	28 ± 0	0.008
ICU-free days	24.2 ± 8.2	27.1 ± 2.7	27.6 ± 1.7	0.047
Hospital stay (days)	27 (23–30)	26 (21–28)	20 (17.8–27.3)	0.004
Death (*n*, %)	2 (1.8%)	0 (0%)	0 (0%)	0.315

Abbreviations: Group A: No vaccination; Group B: One dose of vaccination; Group C: Two doses of vaccination; MODS: multiple organ dysfunction syndrome; ARDS: acute respiratory distress syndrome; AKI: acute kidney injury; NIV: noninvasive ventilation; HFNC: high-flow nasal cannula; MV: mechanical ventilation; CRRT: continuous renal replacement therapy; ECMO: extracorporeal membrane oxygenation; ICU: intensive care unit.

Of the 181 confirmed patients, 111 (61.3%) had comorbidities (e.g., hypertension, diabetes mellitus, and chronic respiratory diseases) before admission. Table 4 demonstrated the differences in the clinical outcome parameters between the vaccinated and no vaccinated patients with or without co-morbidities. In patients with co-morbidities, vaccinated cases had lower incidences of MODS (*P* = 0.015), septic shock (*P* = 0.015), and ARDS (*P* = 0.008). Nevertheless, no significant differences (*P* > 0.1) were discovered in these prognostic variables between the vaccinated and no vaccinated patients without co-morbidities.

**Table 4:** Clinical variables of severity and outcomes in patients with or without co-morbidities.

	**Co-morbidities (*n* = 111)**		***P* value**	**No co-morbidities (*n* = 70)**		***P* value**
	**Vaccinated (*n* = 38)**	**No Vaccinated (*n* = 73)**		**Vaccinated (*n* = 30)**	**No Vaccinated (*n* = 40)**	
MODS (*n*, %)	0 (0%)	11 (15.1%)	0.015	0 (0%)	3 (7.5%)	0.255
Septic shock (*n*, %)	0 (0%)	10 (13.7%)	0.015	0 (0%)	2 (5.0%)	0.503
ARDS (*n*, %)	0 (0%)	12 (16.4%)	0.008	0 (0%)	3 (7.5%)	0.255
Liver injury (*n*, %)	1 (2.6%)	6 (8.2%)	0.419	2 (6.7%)	2 (5.0%)	1.000
AKI (*n*, %)	1 (2.6%)	9 (12.3%)	0.160	0 (0%)	1 (2.5%)	1.000
Cardiac injury (*n*, %)	1 (2.6%)	8 (11.0%)	0.162	0 (0%)	3 (7.5%)	0.255
Thrombo-embolic events (*n*, %)	0 (0%)	0 (0%)	/	0 (0%)	0 (0%)	/
Death (*n*, %)	0 (0%)	2 (2.7%)	0.546	0 (0%)	0 (0%)	/

Abbreviations: MODS: multiple organ dysfunction syndrome; ARDS: acute respiratory distress syndrome; AKI: acute kidney injury.

## DISCUSSION

This clinical retrospective research examined the roles of inactivated SARS-CoV-2 vaccines in older patients with confirmed infection of Delta variant in Nanjing, China. The vaccination rate of older patients was only 37.6% (68/181). We found that patients with two doses of vaccination may have shorter LOS, ICU stay, and respiratory support time, as well as a lower incidence of organ injury and less requirement for supportive treatments. Moreover, in patients with co-morbidities, vaccinated cases had a lower prevalence of ARDS, septic shock, and MODS. However, no difference was found in 28-day mortality across the different groups.

As a serious global epidemic, the COVID-19 is still not alleviated in lots of countries. Apart from traditional isolation and symptomatic treatments, increasing SARS-CoV-2 vaccines were developed to prevent COVID-19. Thompson et al. [19] reported high efficacy of the two-dose messenger RNA (mRNA) vaccines BNT162b2 (Pfizer-BioNTech) and mRNA-1273 (Moderna) for the SARS-CoV-2 infection prevention among adults within the working age. Zhang Y and colleagues [20] investigated the safety, tolerance, and ability to induce an immune response of an inactivated CoronaVac vaccine (Sinovac Life Sciences, Beijing, China) in a phase 1/2 clinical trial in China, and they discovered that two dosages of CoronaVac at varied concentrations and dosage regimens were well tolerable and mildly immunogenic among healthy individuals within the age range of 18–59 years old. Jara’s study [7] also suggests that the CoronaVac vaccination was successful in preventing COVID-19, which resulted in severe sickness and death in Chile. Unfortunately, the efficacy of the vaccines was still questioned by the emerging mutant variants of SARS-CoV-2.

More and more variants have been reported across the globe: Alpha, Beta, Gamma, Delta, Omicron, and so on [6, 21]. In particular, the Delta variant is considered to be a crucial reason for the recurrence or deterioration of the COVID-19 epidemic [5, 6, 22]. After perfectly controlling the epidemic in 2020, China is also suffering from the sporadic outbreak of the COVID-19 (Delta) in 2021. Consequently, it is necessary to investigate the SARS-CoV-2 vaccine efficacy in Delta variants. Lopez and colleagues [4] found that the ChAdOx1 nCoV-19 and BNT162b2 vaccines targeting the Delta variant were effective after the receipt of two vaccine doses. During the Delta strain epidemic in May 2021 in Guangzhou city, China, Li XN and colleagues [23] confirmed the efficacy of two doses of inactivated vaccines in preventing the Delta variant infection in patients (between the ages of 18–59 years). However, the effectiveness of these vaccines in older patients was less investigated.

Wu Z and colleagues confirmed that the CoronaVac was safe and well-tolerated in older adults [10]. Another study also confirmed that the CoronaVac vaccination was successful in the prevention of COVID-19 as well as the associated severe illness and death in older adults [7]. On July 21, 2021, an imported COVID-19 epidemic attributed to the Delta strain was reported in Nanjing city of China [3]. When undertaking our clinical efforts to control the COVID-19 pandemic (Delta variant) in Nanjing, we discovered that there were a number of older and vaccinated patients among confirmed cases. In addition, 61.3% of the older patients had one or more co-morbidities. Therefore, we investigated the roles of Chinese inactivated SARS-CoV-2 vaccines (BBIBP-CorV or CoronaVac) in older patients with confirmed infection of Delta variant, especially in those with co-morbidities in this study. Our results suggested that two doses of the vaccines were effective in improving the disease severity of older patients (aged ≥60 years) with Delta variant, including those with co-morbidities. No difference in 28-day mortality was observed, which might be attributed to the limited sample size employed in this retrospective study.

The immune status and viral load (CT value) of older patients were also investigated in this study. T cell receptor diversity may decline with age in both CD8 and CD4 cells, reducing T cell survival [11]. A study by Thompson MG found that vaccination reduced the load of viral RNA present, the likelihood of febrile manifestations, and the disease duration for those who experienced breakthrough infections despite having received vaccination [19]. The findings of our research demonstrated the differences in immunoglobulin (IgM, IgG) levels in patients with different doses of the vaccines. However, no differences were found in the CD4 or CD8 percentages and the CT values of RT-PCR assays. These results were inconsistent with previous reports, which might attribute to the differences in the days from vaccination to admission [12.5 (8–20) VS. 30 (8–44.5), *P* = 0.035] in our study.

The study had some limitations. It is possible that the results are inconclusive because of the limited sample size and single-center retrospective methodology; hence, large-scale clinical prospective research needs to be carried out to determine the correctness of these findings. Since this research did not employ pathophysiology models and the findings were hypothesis-generating, it is necessary to do more fundamental tests in order to determine the actual processes of vaccinations in older individuals who had suffered from infection with the Delta variant. Finally, because some variables were only collected on admission, the later effects of vaccines on these variables need to be examined in the following clinical studies.

In summary, this clinical retrospective investigation confirmed that the inactivated SARS-CoV-2 vaccines were efficacious in improving the disease severity of older patients with infection of the Delta variant, especially in those with co-morbidities.
